# Supplementary material for: Transcriptomic Analysis of Light-Induced Genes in Nasonia vitripennis: Possible Implications for Circadian Light Entrainment Pathways
Source: Biology (Basel). 2023 Sep 6;12(9):1215. doi: 10.3390/biology12091215 (PMC10525998; doi:10.3390/biology12091215)
Supplement: Supplementary file 1 [file biology-12-01215-s001.zip › supplementary figures.pdf]

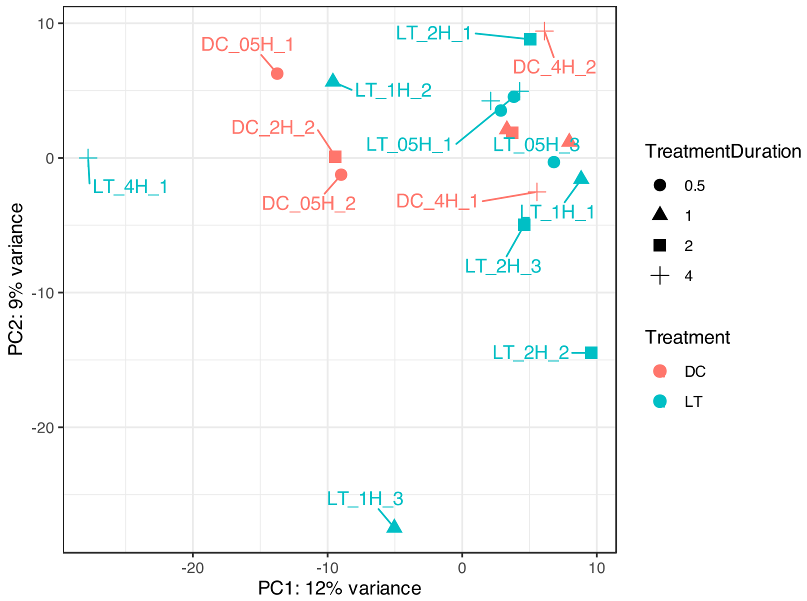

[illegible]
sample names (treatment_treatment duration_replicate) and are colored according to the treatment (DC = dark control, LT = light treatment). Shapes indicate the treatment duration—from 0.5 h to 4 h.

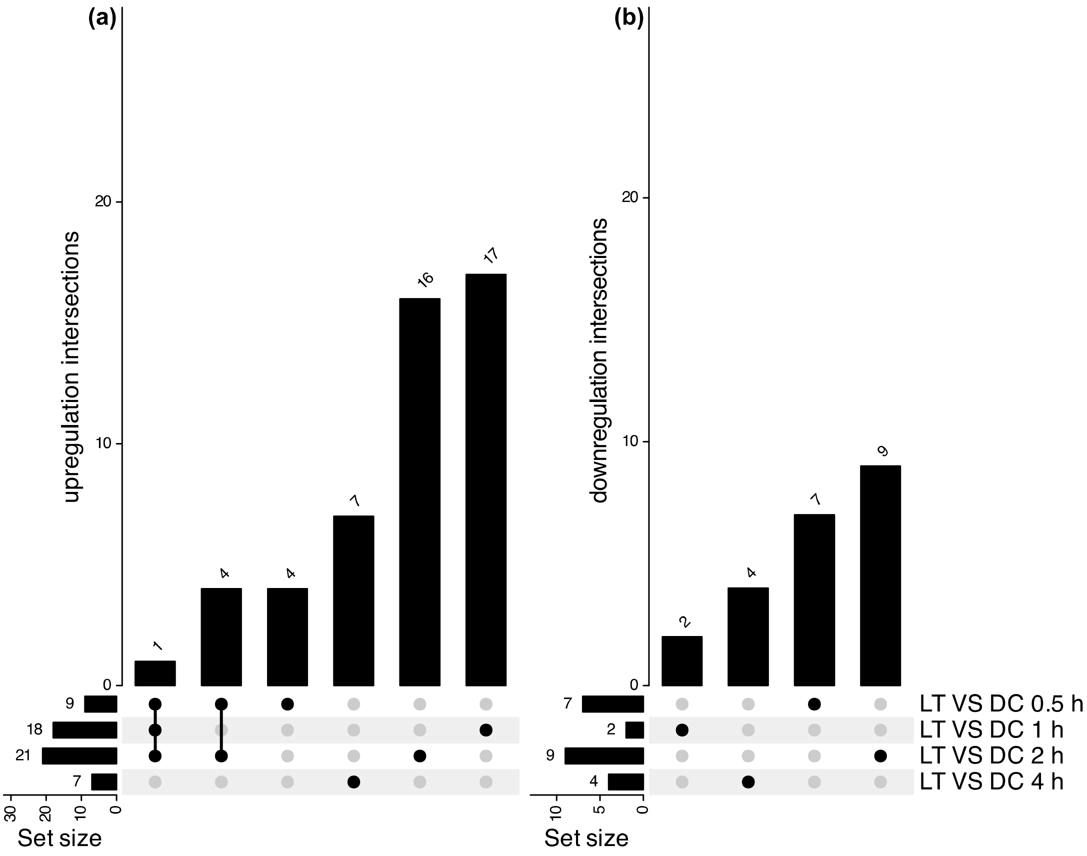

Figure S2. An intersection comparison of the Gene Ontology (GO) enrichment analysis of each time-point comparison presented in Figure 3. The horizontal

bars and numbers indicate the numbers of enriched GO terms in each light–dark comparison. The vertical bars and numbers indicate the similarities and differences in the enriched GO terms between different light–dark comparisons. The vertical black line connects modules that share common GO terms. (a) A comparison of the intersection-enriched GO terms in the upregulated DEGs. (b) The comparison of the intersection-enriched GO terms in the downregulated DEGs.

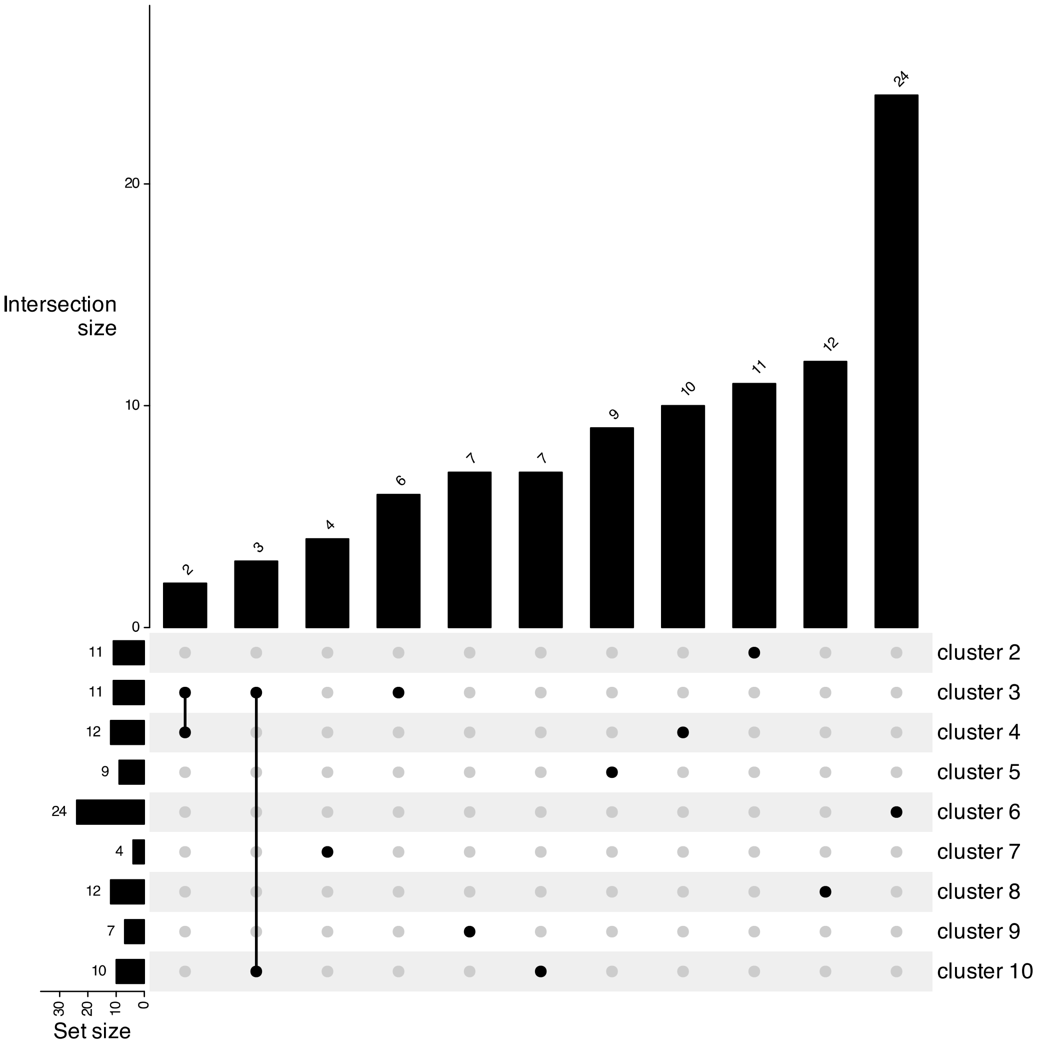

Figure S3. An intersection comparison of the Gene Ontology (GO) enrichment analysis of the time-course clusters presented in Figure 5. The horizontal bars and numbers indicate the numbers of enriched GO terms in each light–dark comparison. The vertical bars and numbers indicate the similarities and differences in the enriched GO terms between different light–dark comparisons. The vertical black line connects modules that shared common GO terms.

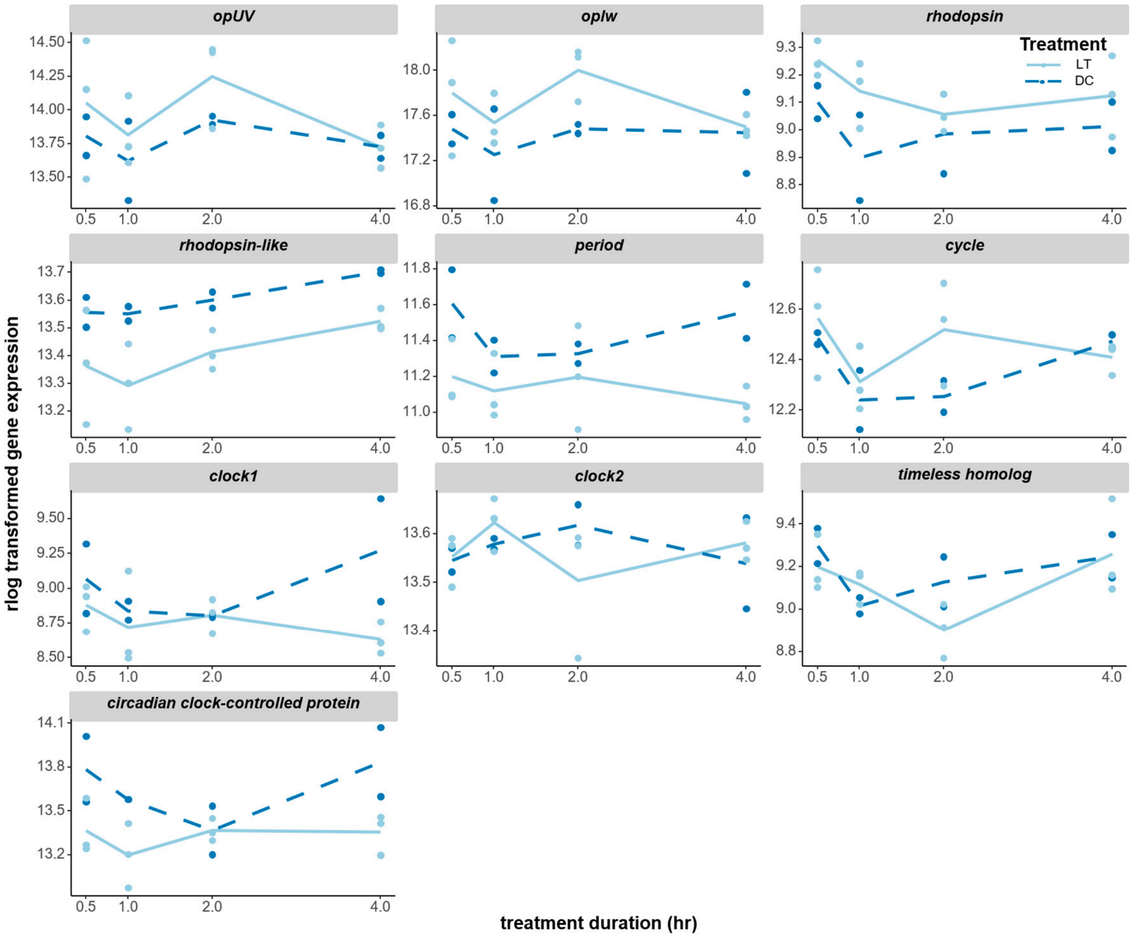

Figure S4. Expression patterns of non-significant opsin and core clock genes. *opUV* (LOC100124184) and *oplw* (*lop1*) encode for UV-sensitive and long-wavelength-sensitive opsins. *rhodopsin* (LOC100122696) and *rhodopsin-like* (LOC100122443) possibly encode for the fourth opsin in *Nasonia*. The core clock genes in *Nasonia* include *period*, *cycle*, *clock1* (LOC100123827), *clock2* (LOC100114103), and *timeless homolog* (LOC100121375). The expression levels of all of these photoreceptor genes, the core clock genes, and a circadian output gene, *circadian clock-controlled protein* (LOC100123404), were not significantly affected by light. The gene expression levels (raw counts) of these DEGs were normalized using rlog data transformation.
